# Neural Modelling in the Exploration of the Biomethane Potential from Cattle Manure: A Case Study on Herds Structure from Wielkopolskie, Podlaskie, and Mazowieckie Voivodeships in Poland

**DOI:** 10.3390/s23010164

**Published:** 2022-12-23

**Authors:** Agnieszka Wawrzyniak, Andrzej Przybylak, Agnieszka Sujak, Piotr Boniecki

**Affiliations:** Department of Biosystems Engineering, Poznan University of Life Sciences, Wojska Polskiego 28, 60-637 Poznan, Poland

**Keywords:** manure, dairy cow herd, artificial neural network, biomethane potential

## Abstract

In the presented study, data on the size and structure of cattle herds in Wielkopolskie, Podlaskie, and Mazowieckie voivodeships in 2019 were analyzed and subjected to modelling with the use of artificial intelligence, namely artificial neural networks (ANNs). The potential amount of biogas (m^3^) from cattle manure and slurry for the analyzed provinces was as follows: for the Mazowieckie Voivodeship, 800,654,186 m^3^; for the Podlaskie voivodeship, 662,655,274 m^3^; and for the Wielkopolskie voivodeship, 657,571,373 m^3^. Neural modelling was applied to find the relationship between the structure of the herds and the amount of generated slurry and manure (biomethane potential), as well as to indicate the most important animal types participating in biogas production. In each of the analyzed cases, the three-layer MLP perceptron with a single hidden layer proved to be the most optimal network structure. Sensitivity analysis of the generated models concerning herd structure showed a significant contribution of dairy cows to the methanogenic potential for both slurry and manure. The amount of slurry produced in the Mazowieckie and Wielkopolskie voivodeships was influenced in turn by heifers (both 6–12 and 12–18 months old) and bulls 12–24 months old, and in the Podlaskie voivodeship by calves and heifers 6–12 months old. As for manure, in addition to cows, bulls 12–24 months old and heifers 12–18 represented the main factor for Mazowieckie and Wielkopolskie voivodeships, and heifers (both 6–12 and 12–18 months old) for Podlaskie voivodeship.

## 1. Introduction

Biomethane plays an important role in meeting the European Union (EU) 2030 greenhouse gas emissions reduction target and will help in achieving net-zero emissions by 2050. Additionally, biomethane increases European energy security by reducing the dependency on Russian natural gas and can alleviate energy cost pressure on households and companies [1].

The 2020 EU natural gas consumption is 400 bcm (billion cubic meters), 155 bcm of which was imported from Russia. Therefore, searching for alternative energy sources is of great importance [1].

Animal manure can be a good raw material for biogas production as an additional energy source for producing heat and/or electricity [2,3,4].

Poland is one of leaders in the European Union in animal breeding [5]. In Poland, in 2019, there were 6358 thousand LSU (Livestock Unit) of cattle including 2461 thousand LSU of cows. Within the polish voivodships, most cattle were raised in: Mazowieckie, with 558.8 thousand heads; Podlaskie, with 457.2 thousand LSU; and Wielkopolskie, with 279.3 thousand LSU [6]. Polish agriculture produced 99 million tons of feces from cows and pigs, including 78 million tons of manure [7]. In comparison, in the European Union and UK, animal farming generated more than 1.4 billion tons of manure annually in the 2016–2019 [8]. Six major countries (Germany, Spain, France, Italy, Poland, United Kingdom) produce about 68% of the total manure, while Ireland (84 million tons) and the Netherlands (73 million tons) also make important contributions [9]. More than 75% of the produced manure comes from cattle, while approximately 12% comes from pigs and chickens [10].

Manure management, i.e., the acquisition, storage, processing, and use of natural fertilizers, affects the profitability of livestock operations and can affect environmental quality [11]. Polish agriculture is a large producer of atmospheric emissions of CH_4_ and N_2_O [12]. Most CH_4_ comes from manure produced by cattle (54%) and pigs (31%). N_2_O emissions from manure management amounted to 9.4 kt in 2019 and have decreased by 32% since 1988, which is mainly related to the decline in livestock populations. In this category, direct emissions account for about 49% and indirect emissions 51% of N_2_O emissions [13].

Among Baltic Sea countries, Poland ranks second in terms of the number of biogas plants, biofuel installed capacity, and pellet plants [14]. It is planned in near future that in Poland troublesome waste in the form of manure and slurry will be disposed of by agricultural biogas plants located mostly near large animal farms [15]. Due to the high cattle population, Poland takes a leading position among the European Union countries in terms of the amount of manure produced. However, its use in this country is primarily limited to fertilizing farmland [16]. Animal manure has a high biomethane potential and cattle manure is reported as the most promising [5].

**Figure 1:**
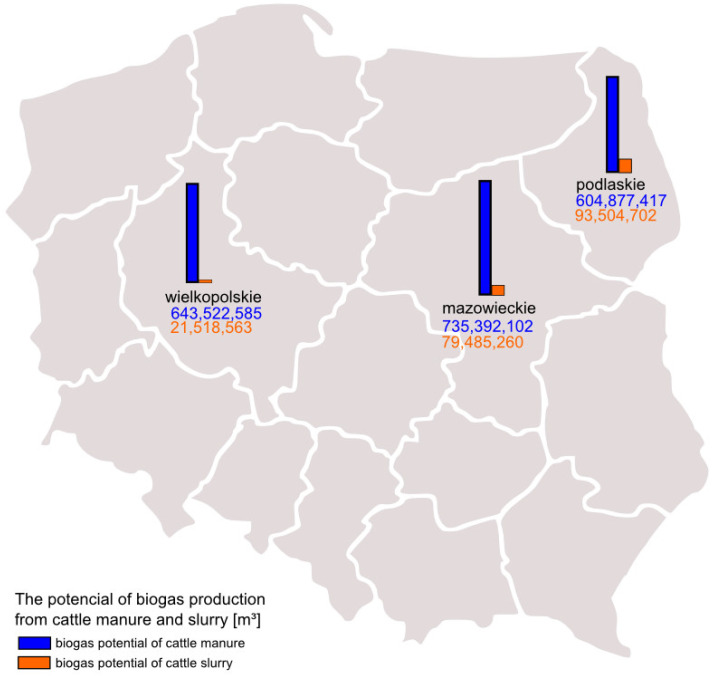
The potential of biogas production from cattle manure and slurry in the examined voivodeships in Poland based on data from 2018 [6].

The first known calculations of the biomethane potential based on cattle excrement data in Poland from 2018 were made in 2021 [7]. The visualization concerning voivodeships included in this study is presented on Figure 1.

The previously published data on possibilities of biogas production in Poland based on the data concerning livestock and poultry population in 2010–2016 indicates Wielkopolskie, Mazowieckie, and Podlaskie voivodeships as the biggest potential biogas producers. However, authors indicated that in order to reach such a potential, the building of biogas plants is required, especially in Podlaskie voivodeship [5].

Estimations of biogas potential in Europe show that up to 41 bcm of biomethane in 2030 and 151 bcm in 2050 could be available. It is predicted that Germany would become the leading producer of biogas in 2030, followed by France and Italy. In the ranking of possible biogas producers, Poland is ranked fifth [1].

In the topic of biogas, neural networks have been used in many aspects, such as biogas yield optimization [17,18] or biogas production prediction [19,20,21]. ANNs have been previously used to model an anaerobic fermentation process [17,18], for biogas production integrated with wastewater purification considering both technological aspects of the process and treated wastewater quality [22], or for biogas production from food, fruits, and vegetables wastes [23], as well as from mixed lignocellulosic co-substrates [21]. ANN model was mainly used defining the optimum region in biogas production [24,25,26]. ANN combined with non-linear regression models were developed to predict the biogas production rate from anaerobic hybrid reactor [20,21,27,28] and as an element of the optimization strategy for biogas production from wastes [24]. Concerning biogas from animal wastes, ANNs were used for the optimization of production from poultry [26] or cattle manure [29,30,31]. ANNs were also used to model and predict the synergistic effect between anaerobic digestion and aerobic process in order to achieve maximum biogas production [32].

To date, there are no publications on the application of artificial intelligence, including artificial neural networks in assessing the impact of cattle herd structure on the amount of biogas extracted from manure or slurry (biomethane potential). Therefore, the aim of the presented research was to conduct a case study of biomethane potential for the Wielkopolskie, Podlaskie, and Mazowieckie voivodeships in Poland. The aim was to find the relationship between the structure of the herds and the amount of the generated slurry and manure (biomethane potential). The data were subjected to neural modelling to indicate the most important animal types participating in biogas production.

## 2. Materials and Methods

### 2.1. Obtaining Data for Modelling

Three voivodeships characterized by the highest number of cattle raised and a high expected biogas potential were selected for the study: Podlaskie, Mazowieckie, and Wielkopolskie voivodeships (see Figure 1). Podlaskie voivodeship, located in the north-eastern part of Poland, covers an area of 2,018,702 km^2^. Mazowieckie voivodeship is the largest in terms of area and population, located in the central and eastern part of Poland which covers an area of 3,555,847 km^2^, and Wielkopolskie voivodeship is located in central-western Poland covering an area of 2,982,650 km^2^.

Data for modeling were obtained through the following procedures [7]:

1. Collection of data from the Agency for Restructuring and Modernization of Agriculture (raw data disclosed by the Agency at the request of A. Wawrzyniak). The data concerned livestock production in rural areas by municipality/province. The data obtained concerned cattle counts in the provinces of Poland selected for the study.
2. Averaging of cattle numbers in province-level data converted to livestock unit (LSU) using conversional factors from the 2010 GUS Agricultural Census [33] dedicated to cattle and specific livestock systems (as in Table 1).
3. Calculation of the amount of waste generated from keeping cattle. Calculations performed separately for manure and for slurry. Calculations were made on the basis of literature data [33].

The following equation was used to calculate amount of slurry:

(1) $$L_{o}=\sum \left(x\times LSU\right)\times (1-\frac{S_{BS}}{S_{s}+S_{BS}})\times O\;(\mathrm{tons})$$

while the equation as below was used for calculation of the amount of manure:

(2) $$L_{G}=\sum \left(x\times LSU\right)\times \left(\frac{S_{BS}}{S_{s}+S_{BS}}\right)\;(m^{3})$$

where:

$L_{o}$: amount of manure (tons)
$L_{G}$: amount of slurry (m^3^)
*x*: cattle population (units)
*LSU*: Livestock Unit. An index of animals per unit according to Regulation of the Council of Ministers of 12 February 2020 [34].
*O*: average amount of manure per year per cattle unit (Mg/LSU·year)
$S_{BS}$ and $S_{s}$ are the conversional factorsbased on animal keeping systems in barns as listed in Table 1.

The amount of obtained biogas was calculated on the basis of [35].

It was assumed for cattle that:

- One ton of manure produces an average of 60 m^3^ of biogas
- 1 m^3^ of slurry produces 28 m^3^ of biogas on average
- The calorific value of biogas is between 19 and 23 (MJ/m^3^)

Therefore, the potential amount of biogas obtained for cattle $P_{b}$ (m^3^) was calculated as follows:

(3) $$P_{b}=\left(L_{G}\cdot P_{G}+L_{o}\cdot P_{G}\right)\;[m^{3}]$$

where:

$P_{G}$: average amount of biogas containing 60% methane from a unit amount of animal feces (m^3^∙(tons or m^3^) ^−1^)
$L_{G}$: estimated amount of slurry (m^3^)
$L_{o}$: estimated amount of manure (tons)

### 2.2. Simulation Studies

Data consisted of 108, 308, and, 213 cases, respectively, for Podlaskie, Mazowieckie, and Wielkopolskie voivodeships. The data were divided into teaching, validating, and test sets in a 2:1:1 ratio, which was 54/27/27, 154/77/77, and 107/53/53/, respectively, for Podlaskie, Mazowieckie, and Wielkopolskie voivodeships. The neural network had 7 independent input variables (input: 1, 2, … …, 7) and 1 output variable (one from O-1 through O-2, either for slurry as for manure for the examined voivodeship) (marked as in Table 2). The structure of the input set was adjusted to the requirements of the ANN simulator implemented in the commercial STATISTICA package (v13.3, Statsoft, Cracow, Poland).

Table 3 shows the exemplary structure of training file for Podlaskie voivodeship.

The ANN simulator was focused on 4 standard ANN topologies: Linear, GRNN (generalized regression neural network), MLP (multi-layer perceptron), and RBF (radial basis function). In all the created networks, the input layer consisted of 7 neurons with a linear PSP (postsynaptic function) and a linear activation function. The hidden layers consisted of a different number of sigmoid neurons, i.e., having a linear PSP function and a logistic activation function. Single sigmoid neuron was obtained as the output of the networks. The generated neural models were trained by the method was BP (back propagation–back propagation of errors) in 5 cycles of 1200 epochs and optimized with the CG (conjugate gradients) algorithm for 200 epochs. In the process of training the network, the Levenberg–Marquardt algorithm was used, tuning the network through 50 epochs. The following parameters were adopted in the learning process with the BP error back propagation algorithm: decreasing learning coefficient (from η = 0.3 to η = 0.03), momentum factor α = 0.4.

The structure and complexity of the ANN (artificial neural network) was determined after several test runs. In our case, 100 networks were tested and 20 were retained. The system automatically assessed the effectiveness of the best network as excellent (under condition that low regression coefficient and correlation approaching to 1 was obtained). The degree of accuracy required for prediction usually varies from application to application. However, roughly, it was assumed that the standard deviation quotient equal to 0.1 (or less) proved a good regression realization by the generated ANN, and above 0.7 (or more) disqualified the generated neural model. Regression statistics were determined independently by standard: for the training and test sets.

## 3. Results

### 3.1. Biomethane Potential in the Analysed Voivodeships

Analysis of the data from the Agency for Restructuring and Modernization of Agriculture in 2013–2018 showed that the structure of livestock was as follows: pigs 64–72%; cattle 27–34%; sheep approximately 1%, and goats less than 1%. The highest percentage of cattle breeding was recorded in 2019 in the Mazowieckie, Podlaskie, and Wielkopolskie voivodeships.

The highest potential for biogas production from cattle manure in Poland in 2019 occurred in the Mazowieckie Voivodeship. The potential amount of biogas (m^3^) from cattle manure and slurry in 2019 for the analyzed provinces was as follows: for the Mazowieckie Voivodeship, 800,654,186 m^3^; for the Podlaskie Voivodeship, 662,655,274 m^3^; and for the Wielkopolskie Voivodeship, 657,571,373 m^3^. Detailed analyses can be found in Supplementary Materials File S1 tables (SF1).

### 3.2. Results on ANN Simulation

**Figure 2:**
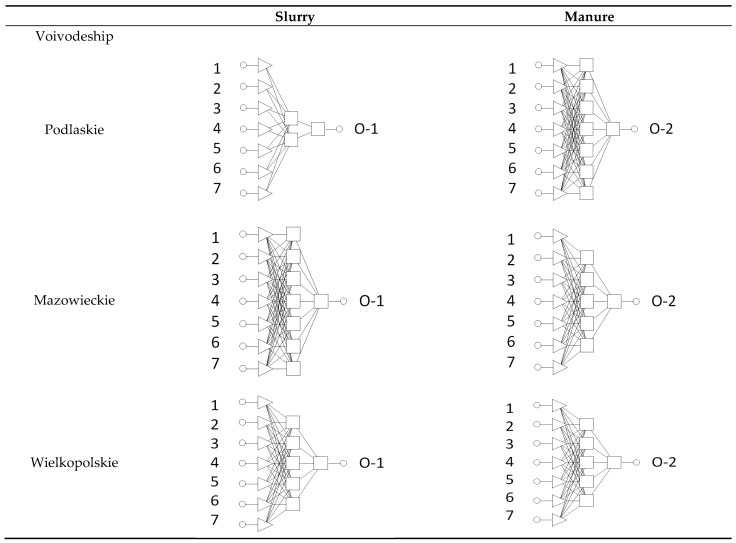
Structures of the generated MPL-type networks obtained for modeling the amounts of slurry or manure in the examined voivodeships, as indicated. Input and output variables are as described in Table 2.

Although The ANN simulator was focused on four different ANN topologies: linear, GRNN (generalized regression neural network), MLP (multi-layer perceptron), and RBF (radial basis function), the generated topologies selected as optimal were ANNs of the MLP (multi-layer perceptron). A graphical representation of the structure of the generated networks is shown in Figure 2 and the regressions statistics of the obtained optimal neural models in Table 4.

Table 5 shows the sensitivity analysis for each of the seven input variables separately for the training and validation files. The compliance of indications for both subfiles is an indicator of the correctness of the assessment of the sensitivity.

Sensitivity is given in the form of three parameters, namely Rank, Error, and Quotient. The key issue in the sensitivity analysis is to define the “importance” of variables. In ANNs, the input variables are ordered according to the loss suffered by the network when the variable is turned off. The basic error is the Error, indicating the quality of the generated ANN in the absence of a given variable. A large value indicates that the network loses a lot without this variable. The quotient is the result of dividing the Error by the error obtained using all the variables. Sensitivity analysis gives an insight into the utility (significance level) of an individual input variable (the lower the variable’s rank, the higher the level of its significance in the generated neural model), indicating variables that (without losing network quality) can be omitted and key variables that should not be omitted.

## 4. Discussion

### 4.1. Biomethane Potential in the Analysed Voivodeships

The largest amount of cattle-derived manure was produced in Wielkopolskie, Podlaskie, and Mazowieckie voivodeships, accounting for 45% of the total manure in Poland. This is in consistence with the previous publication based on data from 2018 year where the same voivodeships were indicated as the largest cattle breeders. The amount of manure and slurry from livestock production depends on the size of livestock farms, and thus the amount of breed animals. Our calculations show that the examined voivodeships produced the most cattle manure, namely 51% of the manure in Poland.

The total production of cattle manure and slurry exceeds 76 million Mg per 2018 year. The apparent leaders included three voivodeships: Mazowieckie, Wielkopolskie, and Podlaskie [7]. Similar studies have been previously performed on data from 2010–2016 by Kozłowski et al. [5].

The highest potential for biogas production from cattle manure in Poland in 2019 occurred in the Mazowieckie Voivodeship. The potential amount of biogas [m^3^] from cattle manure and slurry in 2019 for the analyzed provinces was as follows: for the Mazowieckie Voivodeship, 800,654,186 m^3^; for the Podlaskie Voivodeship, 662,655,274 m^3^; and for the Wielkopolskie Voivodeship, 657,571,373 m^3^. Detailed analyses can be found in Supplementary Materials File S1 tables (SF1).

The obtained values for biogas potential (amount of biogas in m^3^) were lower from that previously calculated for the examined voivodeships, differing by 5.11%, 1.12%, and 1.75%, respectively, for Podlaskie, Wielkopolskie, and Mazowieckie voivodeships. The main reason for the differences in biogas potential compared to the previous evaluation is a gradual decrease in the pig population in Poland along with the increase in the cattle population [34,35,36].

Due to extensive agriculture, Poland is characterized by a large availability of organic waste streams that can be utilized for energy. Unfortunately, due to the lack of stable support for the development of this renewable energy sector in Poland in previous years, as of today, only 123 agricultural biogas plants operate in it (as of December 2022). The annual capacity of these installations for the production of agricultural biogas is approximately 335.335 million m^3^ to generate electricity. In total, 732.645 GWh of electricity was produced from agricultural biogas, of which approximately 607.708 GWh was sold to obliged sellers and other recipients [37].

### 4.2. ANN Simulation

The formulated prediction problem as the effect of a herd structure on the biogas production was best solved by the ANN of the multilayer perceptron type. The conducted analysis allowed for the conclusion that, for the correct prediction of the amount of produced biogas, the knowledge contained in the variables representing the information on the amount of manure produced by cows, heifers, calves, and bulls is sufficient.

Regression analysis of the generated neural models showed that the best predictive ability was achieved by the neural topology of the three-layer perceptron type containing 2–7 neurons in the hidden layer. Variables ranked from 1 to 3 (Table 5) include information on the type of animals that have the greatest impact on the amount of biogas generated. Sensitivity analysis of the model concerning herd structure showed a significant contribution of dairy cows to the methanogenic potential for all the examined voivodeships for both slurry and manure. The amount of slurry produced in the Mazowieckie and Wielkopolskie voivodeships was influenced in turn by heifers (both 6–12 and 12–18 months old) and bulls 12–24 months old, while in the Podlaskie voivodeship by calves and heifers 6–12 months old. As for manure, in addition to cows, bulls 12–24 months old and heifers 12–18 were the main factor for Mazowieckie and Wielkopolskie voivodeships, and heifers (both 6–12 and 12–18 months old) for Podlaskie voivodeship.

For dairy cows, the first 100 days of lactation represent the most demanding period in feeding. Heifers over 12 months of age were another significant factor. This is probably related to the nutritional requirements of this group of animals in the herd: young heifers should consume 6–9 kg of dry matter of feed per day, and the weight gain from protein should already be 0.4 kg greater than its gain from energy. This is the group of animals in the herd with the highest nutritional requirements and therefore one that needs the most intensive feeding [34]. Calves right after birth are fed colostrum and then powdered milk or whey. Feeding animals with forage is common in breeding herds [35].

The only exception in the presented results are early-maturing bulls. This difference may be due to the fact that in young animals the daily weight gain is mainly dominated by meat tissue. The older the animal gets, the more often they deposit fat rather than protein, resulting in an increase in adipose tissue. This results in an increase in energy requirements [36,37,38].

The analysis showed clear differences between Podlaskie and other voivodeships. In addition to the factor related to nutrition, the location of the far-regional factors and the economic situation can have an impact on the obtained results. A common practice of farms in Poland is to arrange feed rations based on the feeds that are available on the farm/region [39,40]. The methanogenic potential in the herd will therefore depend on the category of animals. The method of feeding (depending on the availability of feeds and their price) can be an additional factor. The differing animal keeping systems are also notable.

In a broader perspective, research on the influence of herd structure on the amount of biogas harvested may allow breeders to more carefully select the number and structure (type) of animals. We suspect that the way animals are breeding in the eastern and western parts of Poland may affect the amount of the obtained biogas. This is an issue we intend to study in more detail on data from across Poland.

## 5. Conclusions

The potential amount of biogas (m^3^) from cattle manure and slurry in 2019 for the analyzed provinces was as follows: for the Mazowieckie Voivodeship, 800,654,186 m^3^; for the Podlaskie Voivodeship, 662,655,274 m^3^; and for the Wielkopolskie Voivodeship, 657,571,373 m^3^.

The results of the network sensitivity analysis indicate a significant contribution of dairy cows in the herd structure in maximizing both slurry and manure production. The next categories of animals are heifers of different weight, calves, and bulls.

The obtained research results confirm the that artificial neural networks can be an effective tool in supporting the process of forecasting biogas production from cattle feces based on the structure of the herd.

## Figures and Tables

**Table 1:** The conversial factors $S_{BS}\;\mathrm{and}\;S_{s}$ based on Central Statistical Office of the 2010 Agricultural Census [33].

	Animal Keeping System	
Voivodeship	Stands with a Slatted Floor ($S_{BS}$)	Stands with a Solid Floor ($S_{s}$)
Mazowieckie	0.149	0.851
Podlaskie	0.770	0.230
Wielkopolskie	0.343	0.657

**Table 2:** Input and output variables of created ANNs; values without dimensions were considered in the model.

**Input Variables (Descriptors)-Number of Animals** **of a Certain Type in the Herd Structure**
1 Calves 2 Bulls 6–12 months 3 Bulls 12–24 months 4 Bulls > 24 months 5 Heifers 6–12 months 6 Heifers 12–18 months 7 Dairy Cows months
**Output (options)**
O-1 amount of biogas from slurry for examined voivodeship O-2 amount of biogas from manure for examined voivodeship

**Table 3:** The exemplary structure of training file for Podlaskie voivodeship.

	1	2	3	4	5	6	7	O-1	O-2
No.	Calves DJP	Bulls 6–12 Months	Bulls 12–24 Months	Bulls >24 Months	Heifers 6–12 Months	Heifers 12–18 Months	Dairy Cows	Amount of Biogas from Manure (m^3^)	Amount of Biogas from Slurry (m^3^)
1	410.25	243.3	1012.8	294	545.7	1361.6	13,527	12,653,669	1,956,062
2	396.75	186.9	737.6	238	547.5	1308	13,843	12,466,307	1,927,098
3	174.45	148.8	620	163.8	202.5	476	4511	4,641,118	717,445
4	69.6	66.9	257.6	67.2	100.8	195.2	1792	1,854,335	286,652
5	11.25	13.5	35.2	14	13.5	28.8	382	376,274	58,166
6	286.2	223.5	808	175	338.1	820	8115	7,835,354	1,211,225
7	96.3	87.9	391.2	81.2	106.8	195.2	2195	2,337,194	361,294
8	33	38.4	208	65.8	24.9	76.8	542	749,772	115,903
9	69	49.5	180.8	67.2	102	201.6	2395	2,394,325	370,126
10	67.35	54.3	172.8	72.8	82.5	165.6	2267	2,210,135	341,653
…	…	…	…	…	…	…	…	…	…
108	476.25	257.1	1160	274.4	590.1	1567.2	13,928	13,158,840	2,034,153

**Table 4:** Regression statistics of the obtained optimal neural models. S.D: ratio-quotient of standard deviations determined for errors and for data. Correlation standard: Pearson correlation coefficient between the results given by the generated neural model and the actual output values.

	Learning File	Validation File	Test File	Type of Neural Network
Podlaskie voivodeship				
slurry				
S.D. ratio	0.059180	0.03348	0.095860	MLP: 7-2-1
Correlation	0.998305	0.99954	0.995798	
manure				
S.D. ratio	0.94429	0.02336	0.0616	MLP: 7-7-1
Correlation	0.99902	0.999754	0.998841	
Mazowieckie voivodeship				
slurry				
S.D. ratio	0.01703	0.01429	0.02317	MLP: 7-7-1
Correlation	0.999863	0.9999	0.999816	
manure				
S.D. ratio	0.010581	0.01127	0.01075	MLP: 7-5-1
Correlation	0.999944	0.999945	0.999942	
Wielkopolskie voivodeship				
slurry				
S.D. ratio	0.02645	0.02817	0.02472	MLP: 7-5-1
Correlation	0.999653	0.999605	0.999703	
manure				
S.D. ratio	0.01502	0.008993	0.01077	MLP: 7-5-1
Correlation	0.999887	0.99996	0.999953	

**Table 5:** The sensitivity analysis of the input variables for the examined output variables.

	Calves	Bulls 6–12 Months	Bulls 12–24 Months	Bulls > 24 Months	Heifers 6–12 Months	Heifers 12–18 Months	Dairy Cows
Podlaskie voivodeship							
Slurry-analysis for training set							
Rank	2	5	7	4	3	6	1
Error	782,535.2	331,844.3	234,644.5	402,790.21	456,897.9	315,716.2	2,383,259
Quotient	3.557651	1.508668	1.066768	1.83121	2.077201	1.435345	10.83504
Slurry-analysis for validation set							
Rank	2	5	7	4	3	6	1
Error	641,876.1	244,916.7	131,120.5	288,690.6	350,421.5	168,073.5	2,344,526
Quotient	5.280928	2.015012	1.078772	2.375153	2.883035	1.382797	19.28919
Manure-analysis for training set							
Rank	5	6	4	7	3	2	1
Error	346,955.1	174,304.3	400,851	173,594.9	537,229	557,505.1	2,107,914
Quotient	2.17774	1.094059	2.516029	1.089607	3.372035	3.499303	13.23078
Manure-analysis for validation set							
Rank	5	6	4	7	3	2	1
Error	278,951.5	89,571.02	394,983.2	79,676.33	454,054.6	471,651.7	2,006,711
Quotient	3.609639	1.159051	5.111093	1.031014	5.875479	6.103185	25.96689
Mazowieckie voivodeship							
Slurry-analysis for training set							
Rank	6	7	4	5	2	3	1
Error	103,841.8	69,071.22	199,142.9	164,292.1	212,249.7	205,649	1,830,063
Quotient	2.397802	1.594917	4.598388	3.793654	4.901038	4.748621	42.25781
Slurry-analysis for validation set							
Rank	6	7	4	5	3	2	1
Error	96,951.83	66,217.75	174,988.8	156,794.4	192,573.9	204,318.1	1,696,270
Quotient	2.835091	1.936357	5.117068	4.585022	5.631296	5.974724	49.60278
Manure-analysis for training set							
Rank	4	6	2	5	3	7	1
Error	110,416.7	67,805.55	327,842	106,089.1	200,687	58,909.33	2,127,190
Quotient	3.866067	2.374104	11.47887	3.714542	7.026739	2.062617	74.48018
Manure-analysis for validation set							
Rank	5	6	2	4	3	7	1
Error	85,550.34	67,821.77	337,742.4	112,396.3	177,292.7	50,618.03	1,857,479
Quotient	3.076455	2.438923	12.14547	4.041856	6.37558	1.820263	66.79635
Wielkopolskie voivodeship							
Slurry-analysis for training set							
Rank	6	5	3	4	7	2	1
Error	164,558.3	193,348.8	393,748.6	218,477.6	85,506.16	407,828.6	1,191,321
Quotient	2.608864	3.065301	6.242385	3.463685	1.355592	6.465605	18.88689
Slurry-analysis for validation set							
Rank	6	5	2	4	7	3	1
Error	154,924.7	199,205.6	361,707.1	212,250.6	83,400.45	357,936.4	1,010,796
Quotient	2.609011	3.354724	6.091332	3.574408	1.404506	6.027831	17.02231
Manure-analysis for training set							
Rank	6	5	2	4	7	3	1
Error	126,985.5	149,031.5	450,806.2	165,423.2	79,804.95	209,430.4	1,260,193
Quotient	3.629729	4.259889	12.88576	4.728427	2.28113	5.98632	36.02112
Manure-analysis for validation set							
Rank	6	5	2	4	7	3	1
Error	121,494.7	158,065.2	514,812.5	184,429.8	78,210.54	261,297	1,466,372
Quotient	4.614739	6.003798	19.55415	7.005207	2.970675	9.924857	55.69727

## Data Availability

Data available on request.

## Supplementary Materials

The following supporting information can be downloaded at: https://www.mdpi.com/article/10.3390/s23010164/s1, File S1: Detailed data on cattle number and herd structure for Wielkopolskie, Podlaskie and Mazowieckie voivodeships in Poland (including individual districts).
