# Supplementary material for: Neural Modelling in the Exploration of the Biomethane Potential from Cattle Manure: A Case Study on Herds Structure from Wielkopolskie, Podlaskie, and Mazowieckie Voivodeships in Poland
Source: Sensors (Basel). 2022 Dec 23;23(1):164. doi: 10.3390/s23010164 (PMC9824757; doi:10.3390/s23010164)
Supplement: Supplementary file 1 [file sensors-23-00164-s001.zip › sensors-2064485-supplementary.pdf]

Mazowieckie Voivodeship												
**Community**	**District**	**Calves**	**Bulls 6-12 months**	**Bulls 12-24 months**	**Bulls >24 months**	**Heifers 6-12 months**	**Heifers 12-18**	**Dairy cows**	**Manure mass (tons)**	**Slurry volume (m^3)**	**Amount of biogas from manure (m^3)**	**Amount of biogas from slurry (m^3)**
białobrzeski	Białobrzegi	71	68	73	22	27	23	234	4866	1127	291959	31557
białobrzeski	Promna	75	84	110	152	24	30	298	8517	1973	511034	55235
białobrzeski	Radzanów	187	238	273	73	55	42	519	12641	2928	758480	81981
białobrzeski	Stara Błotnica	615	510	809	140	237	165	1641	37478	8680	2248679	243050
białobrzeski	Stromiec	267	281	284	112	98	103	1015	20897	4840	1253833	135521
białobrzeski	Wyśmierzyce	141	95	149	124	51	31	416	10205	2364	612328	66184
ciechanowski	Ciechanów	998	541	897	144	561	457	3186	63275	14655	3796503	410347
ciechanowski	Glinojeck	389	221	460	72	212	177	1291	26706	6185	1602344	173190
ciechanowski	Gołymin-Ośrodek	871	470	879	169	508	497	2905	59637	13813	3578234	386755
ciechanowski	Grudusk	799	372	616	138	495	427	3092	57504	13319	3450255	372923
ciechanowski	Ojrzeń	816	425	576	120	451	436	2876	54172	12547	3250312	351312
ciechanowski	Opinogóra Górna	1585	670	1301	225	847	880	5942	111118	25736	6667058	720613
ciechanowski	Regimin	660	282	444	82	356	341	2325	42918	9940	2575068	278328
ciechanowski	Sońsk	1433	1016	1668	250	750	738	4700	98656	22850	5919351	639796
garwoliński	Borowie	520	193	356	66	307	280	2158	38176	8842	2290585	247579
garwoliński	Garwolin	213	98	159	108	120	122	818	16503	3822	990207	107027
garwoliński	Górzno	394	146	230	74	232	231	1506	27489	6367	1649310	178267
garwoliński	Łaskarzew	410	184	270	85	211	198	1536	28236	6540	1694168	183115
garwoliński	Maciejowice	765	438	699	183	429	307	2499	50287	11647	3017243	326120
garwoliński	Miastków Kościelny	567	227	300	132	299	331	2185	39841	9228	2390461	258374
garwoliński	Parysów	264	126	170	47	159	146	1076	19422	4498	1165306	125953

garwoliński	Pilawa	121	73	107	31	55	41	319	6867	1591	412040	44536
garwoliński	Sobolew	579	421	506	128	282	237	1699	35405	8200	2124304	229607
garwoliński	Trojanów	1279	679	989	277	643	587	4730	89039	20622	5342334	577429
garwoliński	Wilga	76	16	30	10	26	47	320	5362	1242	321749	34776
garwoliński	Żelechów	627	344	493	133	303	325	2328	44177	10232	2650636	286495
gostyniński	Gostynin	530	276	464	120	264	281	1535	32468	7520	1948055	210557
gostyniński	Pacyna	737	442	889	128	282	296	2284	47784	11067	2867027	309884
gostyniński	Sanniki	425	257	454	142	191	164	1247	27328	6330	1639687	177226
gostyniński	Szczawin Kościelny	566	299	512	89	239	226	1432	30586	7084	1835182	198357
grodziski	Baranów	258	128	196	108	161	135	1499	26076	6040	1564579	169108
grodziski	Grodzisk Mazowiecki	26	22	21	43	40	24	287	5185	1201	311091	33624
grodziski	Jaktorów	56	31	56	50	29	42	330	6451	1494	387081	41838
grodziski	Milanówek	5	0	1	2	2	1	14	252	58	15144	1637
grodziski	Żabia Wola	37	29	30	66	29	15	250	5129	1188	307755	33264
grójecki	Belsk Duży	0	0	0	3	0	0	13	220	51	13189	1426
grójecki	Błędów	21	11	17	8	9	9	141	2328	539	139673	15097
grójecki	Chynów	58	78	176	76	16	17	236	6821	1580	409241	44233
grójecki	Goszczyn	5	1	2	14	2	1	49	928	215	55708	6021
grójecki	Grójec	17	52	29	26	8	8	110	2512	582	150715	16290
grójecki	Jasieniec	19	16	27	14	12	5	158	2741	635	164440	17774
grójecki	Mogielnica	52	70	75	39	26	10	245	5166	1196	309941	33500
grójecki	Nowe Miasto nad Pilicą	293	166	275	93	88	82	909	18466	4277	1107988	119757
grójecki	Pniewy	22	11	8	42	9	13	114	2542	589	152517	16485
grójecki	Warka	10	4	4	14	12	2	125	1990	461	119391	12904
kozienicki	Garbatka-Letnisko	39	29	41	11	49	34	250	4532	1050	271946	29393
kozienicki	Głowaczów	337	289	359	179	148	121	1153	25167	5829	1510021	163211
kozienicki	Gniewoszów	281	300	516	105	200	139	887	22367	5180	1342015	145052
kozienicki	Grabów nad Pilicą	237	174	208	95	161	111	1135	21205	4911	1272313	137519
kozienicki	Kozienice	89	131	110	48	63	29	377	8012	1856	480745	51962

kozienicki	Magnuszew	313	210	299	104	124	93	661	16197	3751	971804	105038
kozienicki	Sieciechów	138	100	157	44	50	56	366	8482	1965	508925	55007
legionowski	Jabłonna	0	0	0	2	0	1	1	59	14	3527	381
legionowski	Legionowo	0	0	0	0	0	0	1	13	3	767	83
legionowski	Nieporęt	99	42	111	46	20	13	203	5113	1184	306758	33156
legionowski	Serock	125	90	167	42	38	52	334	7989	1850	479365	51812
legionowski	Wieliszew	33	34	51	26	14	9	109	2719	630	163137	17633
lipski	Chotcza	129	96	94	62	67	46	598	11055	2561	663320	71695
lipski	Ciepielów	609	322	535	177	357	333	2578	48759	11293	2925534	316208
lipski	Lipsko	73	45	56	69	41	25	457	8373	1939	502369	54299
lipski	Rzeczniów	134	136	185	132	77	52	678	14523	3364	871392	94185
lipski	Sienno	135	167	208	98	118	104	678	14960	3465	897578	97015
lipski	Solec nad Wisłą	97	43	100	128	62	40	645	12553	2907	753189	81409
łosicki	Huszlew	831	673	1149	163	222	209	905	33391	7734	2003457	216545
łosicki	Łosice	570	484	749	79	237	194	1165	29800	6902	1788024	193260
łosicki	Olszanka	1176	726	1012	59	498	540	3407	67412	15613	4044717	437175
łosicki	Platerów	472	378	664	78	200	154	737	22299	5165	1337913	144609
łosicki	Sarnaki	434	285	518	66	178	183	1036	24195	5604	1451706	156908
łosicki	Stara Kornica	1078	887	1522	163	341	292	1703	50002	11581	3000105	324268
makowski	Czerwonka	650	502	806	140	346	287	1902	42485	9840	2549073	275518
makowski	Karniewo	2008	1022	1470	260	1021	955	5945	117104	27123	7026265	759438
makowski	Krasnosielc	1398	499	706	156	1146	1103	7953	131913	30553	7914756	855471
makowski	Maków Mazowiecki	15	22	64	2	20	13	70	1907	442	114445	12370
makowski	Młynarze	435	309	399	59	289	226	1786	33397	7735	2003840	216586
makowski	Płoniawy-Bramura	1322	711	980	161	887	758	5299	97032	22474	5821929	629266
makowski	Różan	298	203	343	110	146	109	907	20090	4653	1205410	130287
makowski	Rzewnie	631	342	560	162	401	297	2527	48014	11121	2880829	311376
makowski	Sypniewo	1335	612	954	182	932	804	6403	111539	25834	6692362	723348
makowski	Szelków	893	443	871	165	375	378	2446	51830	12004	3109796	336124

miński	Cegłów	223	121	178	87	109	121	920	17680	4095	1060829	114660
miński	Dębe Wielkie	99	73	88	148	47	57	516	11375	2635	682490	73767
miński	Dobre	327	172	237	174	173	163	1313	25933	6006	1555952	168176
miński	Halinów	50	17	43	78	27	19	250	5489	1271	329341	35597
miński	Jakubów	322	179	426	249	169	201	1437	31182	7222	1870915	202219
miński	Kałuszyn	274	151	293	125	144	130	1141	22800	5281	1367971	147858
miński	Latowicz	1159	435	696	277	673	735	5539	96845	22430	5810695	628052
miński	Mińsk Mazowiecki	261	90	149	145	156	125	1159	21651	5015	1299074	140411
miński	Mrozy	398	116	224	131	257	196	1764	31375	7267	1882494	203470
miński	Siennica	722	295	356	194	401	432	3165	56029	12977	3361728	363354
miński	Stanisławów	208	164	231	188	136	104	826	18894	4376	1133637	122530
miński	Sulejówek	2	0	1	2	0	0	7	139	32	8358	903
mławski	Dzierzgowo	1101	530	656	271	832	705	6105	104118	24115	6247081	675219
mławski	Lipowiec Kościelny	625	213	361	99	397	344	2883	49361	11433	2961650	320112
mławski	Mława	42	33	27	36	31	5	89	2435	564	146075	15789
mławski	Radzanów	850	277	434	224	653	610	5832	94410	21866	5664582	612259
mławski	Strzegowo	1469	669	1010	456	980	812	7147	127264	29476	7635833	825323
mławski	Stupsk	842	318	556	209	508	519	4351	75117	17398	4507020	487144
mławski	Szreńsk	891	329	390	268	735	588	5510	90999	21077	5459961	590143
mławski	Szydłowo	749	312	514	264	509	408	3894	68499	15865	4109933	444224
mławski	Wieczfnia Kościelna	842	477	683	294	667	493	5156	89178	20655	5350654	578328
mławski	Wiśniewo	579	206	268	143	416	341	2813	48230	11171	2893788	312777
nowodworski	Czosnów	20	38	35	22	32	11	187	3561	825	213630	23090
nowodworski	Leoncin	34	21	26	15	33	11	151	2849	660	170920	18474
nowodworski	Nasielsk	827	639	1042	203	436	328	2307	52829	12236	3169760	342605
nowodworski	Nowy Dwór Mazowiecki	0	0	0	0	0	0	0	0	0	0	0
nowodworski	Pomiechówek	116	121	192	49	62	36	212	6841	1584	410468	44366
nowodworski	Zakroczym	14	19	37	18	10	4	72	1799	417	107965	11670
ostrołęcki	Baranowo	1074	283	323	386	1227	791	10351	158430	36694	9505790	1027438

ostrołęcki	Czarnia	428	189	194	125	607	260	4469	67864	15718	4071861	440109
ostrołęcki	Czerwin	2099	703	1065	592	1798	1518	14700	238479	55235	14308756	1546570
ostrołęcki	Goworowo	1530	785	1207	448	965	760	7044	127791	29598	7667463	828742
ostrołęcki	Kadzidło	1298	343	394	321	1593	898	12640	190403	44100	11424170	1234788
ostrołęcki	Lelis	709	219	325	276	924	540	6240	99271	22992	5956234	643783
ostrołęcki	Łyse	1463	354	408	400	1976	991	15286	228553	52936	13713183	1482197
ostrołęcki	Myszyniec	1098	458	548	428	1722	824	13702	207260	48004	12435579	1344106
ostrołęcki	Olszewo-Borki	621	244	308	208	584	369	4319	70205	16260	4212301	455289
ostrołęcki	Rzekuń	714	322	534	226	466	468	3726	66296	15355	3977775	429940
ostrołęcki	Troszyn	1693	449	667	380	1217	1187	10125	164785	38166	9887081	1068651
Ostrołęka	Ostrołęka	6	2	12	8	6	4	62	1141	264	68475	7401
ostrowski	Andrzejewo	2260	756	1345	327	1387	1420	10483	180641	41839	10838488	1171484
ostrowski	Boguty-Pianki	1273	343	610	184	924	860	7231	118032	27338	7081896	765451
ostrowski	Brok	80	44	73	61	48	34	468	8673	2009	520350	56242
ostrowski	Małkinia Górna	360	190	323	110	195	188	1372	26893	6229	1613577	174404
ostrowski	Nur	918	365	638	178	576	576	4607	79842	18492	4790506	517785
ostrowski	Ostrów Mazowiecka	1191	699	1189	322	639	512	4536	88535	20506	5312122	574164
ostrowski	Stary Lubotyń	1752	507	733	228	1175	1021	8807	144373	33439	8662386	936279
ostrowski	Szulborze Wielkie	664	255	387	114	392	431	2989	52356	12126	3141350	339534
ostrowski	Wąsewo	814	404	560	225	500	501	3556	65345	15135	3920725	423774
ostrowski	Zaręby Kościelne	776	348	590	230	477	433	3639	65731	15224	3943882	426277
otwocki	Celestynów	42	26	42	40	19	9	154	3458	801	207496	22427
otwocki	Józefów	0	0	0	5	0	0	2	115	27	6901	746
otwocki	Karczew	15	10	18	73	15	8	116	3179	736	190742	20616
otwocki	Kołbiel	280	197	239	173	98	126	725	17760	4114	1065622	115178
otwocki	Osieck	92	64	68	47	44	32	373	7221	1672	433242	46827
otwocki	Otwock	0	0	0	4	0	0	2	97	22	5828	630
otwocki	Sobienie-Jeziory	98	36	38	41	26	28	320	5924	1372	355412	38415
otwocki	Wiązowna	90	64	100	93	45	31	412	8859	2052	531546	57452

piaseczyński	Góra Kalwaria	9	3	7	8	10	6	77	1327	307	79632	8607
piaseczyński	Konstancin-Jeziorna	102	3	4	12	83	101	438	7411	1717	444667	48062
piaseczyński	Lesznowola	67	12	43	21	33	37	262	4843	1122	290579	31407
piaseczyński	Piaseczno	22	14	28	51	15	63	226	4885	1131	293071	31677
piaseczyński	Prażmów	147	81	104	71	44	43	450	9285	2151	557119	60216
piaseczyński	Tarczyn	25	37	89	106	16	11	234	6161	1427	369636	39952
Płock	Płock	58	97	206	10	47	19	119	4663	1080	279805	30243
płocki	Bielsk	787	419	677	142	380	374	2973	55853	12936	3351184	362215
płocki	Bodzanów	622	350	592	160	274	316	1997	41253	9555	2475154	267528
płocki	Brudzeń Duży	799	505	997	178	326	343	1998	47137	10918	2828227	305690
płocki	Bulkowo	721	459	686	143	417	422	2923	55983	12966	3359006	363060
płocki	Drobin	1289	423	753	141	775	757	5547	95916	22215	5754949	622027
płocki	Gąbin	314	285	442	104	120	130	814	20267	4694	1215991	131431
płocki	Łąck	67	30	33	27	12	18	108	2674	619	160453	17343
płocki	Mała Wieś	747	449	944	163	302	357	2228	49003	11350	2940180	317791
płocki	Nowy Duninów	50	31	43	9	20	32	169	3379	783	202742	21913
płocki	Radzanowo	522	387	545	119	287	253	1662	35113	8133	2106783	227713
płocki	Słubice	313	230	463	119	104	72	567	16726	3874	1003550	108469
płocki	Słupno	245	197	285	47	98	75	564	13330	3087	799811	86448
płocki	Stara Biała	582	380	738	118	168	142	1095	28319	6559	1699152	183654
płocki	Staroźreby	770	410	577	133	437	442	3072	56782	13151	3406892	368236
płocki	Wyszogród	508	266	478	118	265	253	1770	35215	8156	2112917	228376
płoński	Baboszewo	1089	433	639	155	649	681	4467	79593	18435	4775592	516172
płoński	Czerwińsk	0	0	3	0	0	0	0	31	7	1840	199
płoński	Czerwińsk nad Wisłą	248	172	329	99	93	103	688	16472	3815	988329	106824
płoński	Dzierzążnia	669	348	492	128	406	365	2762	50524	11702	3031429	327654
płoński	Joniec	163	131	148	33	91	50	397	8852	2050	531124	57407
płoński	Naruszewo	879	356	623	148	492	484	2908	56066	12986	3363990	363599
płoński	Nowe Miasto	291	187	248	117	156	152	961	20337	4710	1220247	131891

płoński	Płońsk	496	230	332	152	332	289	2209	40405	9358	2424315	262033
płoński	Raciąż	2197	823	1235	240	1391	1302	9958	170196	39419	10211744	1103742
płoński	Sochocin	511	280	447	142	279	258	1855	36578	8472	2194697	237215
płoński	Załuski	157	156	251	105	61	50	270	9540	2209	572378	61866
pruszkowski	Brwinów	0	1	0	9	2	1	39	681	158	40870	4418
pruszkowski	Michałowice	5	0	0	5	0	0	16	304	70	18212	1968
pruszkowski	Nadarzyn	16	18	35	69	5	3	96	2969	688	178128	19253
pruszkowski	Piastów	0	0	0	0	0	0	0	0	0	0	0
pruszkowski	Pruszków	0	0	0	0	0	0	3	38	9	2300	249
pruszkowski	Raszyn	22	2	19	7	23	31	176	3024	700	181425	19609
przasnyski	Chorzele	1412	551	583	533	1975	987	13778	214062	49579	12843747	1388223
przasnyski	Czernice Borowe	1280	476	589	643	867	776	6700	118689	27490	7121348	769715
przasnyski	Jednorożec	820	283	367	361	991	553	7304	115667	26790	6940000	750114
przasnyski	Krasne	1117	722	1053	215	466	441	3015	64349	14904	3860953	417313
przasnyski	Krzynowłoga Mała	1322	566	819	431	1062	800	8504	141721	32824	8503275	919081
przasnyski	Przasnysz	2114	1061	1413	553	1338	1399	10550	186723	43247	11203408	1210927
przysuski	Borkowice	54	33	37	22	29	24	250	4554	1055	273211	29530
przysuski	Gielniów	70	43	76	45	21	26	322	6343	1469	380563	41133
przysuski	Klwów	96	76	101	118	49	48	552	11352	2629	681148	73622
przysuski	Odrzywół	184	146	180	102	91	57	796	15682	3632	940940	101702
przysuski	Potworów	241	111	253	111	106	100	1009	19784	4582	1187045	128302
przysuski	Przysucha	89	64	75	69	39	23	366	7479	1732	448770	48506
przysuski	Rusinów	175	83	164	120	96	75	894	17038	3946	1022259	110491
przysuski	Wieniawa	294	291	330	172	103	82	1072	23064	5342	1383844	149574
pułtuski	Gzy	841	427	679	164	477	476	3464	64091	14844	3845464	415639
pułtuski	Obryte	642	367	615	170	403	296	2673	50700	11743	3041972	328793
pułtuski	Pokrzywnica	472	338	548	128	330	253	1966	39071	9049	2344261	253381
pułtuski	Pułtusk	529	299	539	98	321	256	1657	34449	7979	2066948	223407
pułtuski	Świercze	700	338	590	109	376	365	3111	55552	12867	3333126	360263

pułtuski	Winnica	743	459	769	145	475	414	3064	58853	13631	3531152	381667
pułtuski	Zatory	644	537	1121	298	331	249	1535	43518	10079	2611107	282223
Radom	Radom	56	50	52	29	29	17	175	3871	897	232264	25104
radomski	Gózd	367	246	383	103	181	184	1336	27055	6266	1623277	175453
radomski	Iłża	246	358	397	137	122	102	891	21252	4922	1275112	137821
radomski	Jastrzębia	320	240	312	99	162	112	961	20543	4758	1232554	133221
radomski	Jedlińsk	595	471	641	208	225	186	1680	37456	8675	2247376	242909
radomski	Jedlnia-Letnisko	234	195	279	68	92	50	409	11356	2630	681378	73647
radomski	Kowala	108	120	82	71	67	29	462	9234	2139	554013	59881
radomski	Pionki	223	118	227	115	91	121	700	15790	3657	947420	102402
radomski	Przytyk	430	302	470	259	190	149	1400	31565	7311	1893919	204705
radomski	Skaryszew	888	501	742	278	575	462	3978	73950	17128	4437012	479577
radomski	Wierzbica	451	276	289	100	230	181	1633	30269	7011	1816127	196297
radomski	Wolanów	165	140	188	69	89	71	701	14036	3251	842138	91023
radomski	Zakrzew	210	197	267	135	93	73	672	15994	3704	959650	103724
Siedlce	Siedlce	0	2	0	0	0	0	8	110	25	6594	713
siedlecki	Domanice	158	100	162	37	103	61	618	11921	2761	715271	77310
siedlecki	Korczew	333	266	394	91	159	127	974	21670	5019	1300224	140535
siedlecki	Kotuń	594	259	340	129	272	268	2278	40812	9452	2448699	264669
siedlecki	Mokobody	1269	420	715	126	798	800	6061	102306	23695	6138349	663467
siedlecki	Mordy	1154	528	783	136	582	571	3874	72254	16735	4335257	468579
siedlecki	Paprotnia	915	654	1099	120	395	381	2448	54340	12586	3260395	352402
siedlecki	Przesmyki	753	394	660	120	310	328	2033	42373	9814	2542364	274793
siedlecki	Siedlce	202	157	208	88	86	86	552	12954	3000	777228	84007
siedlecki	Skórzec	360	228	408	121	141	138	981	22389	5186	1343357	145197
siedlecki	Suchożebry	853	450	691	120	360	481	2880	55677	12895	3340603	361071
siedlecki	Wiśniew	880	535	898	220	329	410	2387	52815	12232	3168878	342510
siedlecki	Wodynie	355	195	316	109	203	181	1376	26823	6213	1609398	173953
siedlecki	Zbuczyn	1040	733	1340	149	431	337	2250	55023	12744	3301381	356832

siedlecki	Zbuczyn Poduchowny	906	430	839	172	418	429	3118	60878	14100	3652652	394799
sierpecki	Gozdowo	1112	548	789	174	611	504	3564	68456	15855	4107364	443947
sierpecki	Mochowo	590	386	578	169	312	246	1585	35512	8225	2130707	230299
sierpecki	Rościszewo	1255	600	1002	200	696	613	4261	81920	18974	4915226	531265
sierpecki	Sierpc	876	652	1060	175	436	376	2642	57428	13301	3445692	372430
sierpecki	Szczutowo	747	386	422	207	507	426	3326	59736	13835	3584138	387394
sierpecki	Zawidz	2492	904	1414	373	1567	1332	11819	200047	46333	12002797	1297329
sochaczewski	Brochów	59	48	70	76	33	48	324	7131	1652	427836	46243
sochaczewski	Iłów	180	201	422	123	105	85	630	16954	3927	1017237	109949
sochaczewski	Młodzieszyn	225	154	253	143	156	117	802	18211	4218	1092652	118100
sochaczewski	Nowa Sucha	289	150	226	109	188	168	1344	25005	5791	1500283	162159
sochaczewski	Rybno	221	132	195	137	130	104	868	18029	4176	1081763	116923
sochaczewski	Sochaczew	198	89	196	137	80	83	607	14089	3263	845320	91367
sochaczewski	Teresin	103	37	62	40	43	51	425	7807	1808	468400	50627
sokołowski	Bielany	2106	689	1072	228	956	981	7292	128605	29786	7716308	834021
sokołowski	Ceranów	601	245	371	90	376	331	2792	48002	11118	2880139	311301
sokołowski	Jabłonna Lacka	826	520	860	208	383	470	2686	56692	13131	3401525	367656
sokołowski	Kosów Lacki	999	493	754	190	652	652	5039	88478	20493	5308671	573791
sokołowski	Repki	1770	899	1326	204	960	965	6341	118632	27476	7117898	769342
sokołowski	Sabnie	413	364	418	132	156	164	1245	27009	6256	1620517	175154
sokołowski	Sokołów Podlaski	960	498	722	222	545	581	3320	65563	15185	3933761	425183
sokołowski	Sterdyń	1017	402	634	171	644	676	4785	83565	19355	5013914	541932
szydłowiecki	Chlewiska	45	38	42	30	15	12	195	3870	896	232225	25100
szydłowiecki	Jastrząb	89	34	28	18	39	43	433	7032	1629	421932	45605
szydłowiecki	Mirów	62	74	82	51	49	33	509	9184	2127	551022	59558
szydłowiecki	Orońsko	141	129	161	67	51	44	441	9891	2291	593465	64145
szydłowiecki	Szydłowiec	72	44	48	42	40	16	321	5968	1382	358096	38705
Warszawa	Białołęka	0	0	0	0	0	0	0	0	0	0	0
Warszawa	Targówek	0	0	0	0	0	0	1	13	3	767	83

Warszawa	Ursynów	0	0	0	0	0	0	0	0	0	0	0
Warszawa	Ursynów dzielnica	0	0	0	0	0	0	0	0	0	0	0
Warszawa	Warszawa	1	0	1	32	0	1	41	1119	259	67133	7256
Warszawa	Wawer	0	0	0	0	0	0	1	13	3	767	83
warszawski zachodni	Błonie	101	11	2	42	50	79	460	7886	1826	473154	51141
warszawski zachodni	Izabelin	0	0	0	4	0	0	1	84	20	5061	547
warszawski zachodni	Kampinos	101	56	85	60	56	46	392	8046	1863	482739	52177
warszawski zachodni	Leszno	132	82	112	52	53	46	461	9208	2133	552479	59715
warszawski zachodni	Łomianki	4	1	3	7	3	0	27	524	121	31439	3398
warszawski zachodni	Ożarów Mazowiecki	32	20	11	7	37	13	197	3168	734	190090	20546
warszawski zachodni	Stare Babice	11	1	2	12	0	0	18	490	114	29407	3178
węgrowski	Grębków	1144	522	688	165	836	695	5443	94053	21784	5643188	609947
węgrowski	Korytnica	1278	716	938	257	737	674	5387	97946	22685	5876755	635192
węgrowski	Liw	1175	510	762	182	688	586	4840	85739	19858	5144346	556029
węgrowski	Łochów	119	83	107	114	98	76	758	14520	3363	871200	94164
węgrowski	Miedzna	819	400	601	100	435	418	3008	55421	12836	3325267	359413
węgrowski	Sadowne	346	127	220	329	319	241	2580	45945	10641	2756723	297962
węgrowski	Stoczek	335	221	275	121	267	172	1939	34029	7881	2041720	220680
węgrowski	Węgrów	174	58	83	27	90	91	730	12492	2893	749547	81015
węgrowski	Wierzbno	623	399	482	134	330	322	2453	45956	10644	2757374	298032
wołomiński	Dąbrówka	178	119	196	132	132	129	1246	22912	5307	1374719	148587
wołomiński	Jadów	223	80	112	176	139	115	1230	22456	5201	1347383	145633
wołomiński	Klembów	281	153	183	179	152	154	1275	24651	5709	1479042	159863

wołomiński	Kobyłka	0	0	0	0	0	0	7	89	21	5368	580
wołomiński	Marki	0	0	0	0	0	0	1	13	3	767	83
wołomiński	Poświętne	196	152	175	248	131	94	1166	23550	5454	1412982	152723
wołomiński	Radzymin	98	47	81	156	97	58	763	14703	3405	882203	95353
wołomiński	Strachówka	231	136	165	125	128	113	1211	22010	5098	1320621	142740
wołomiński	Tłuszcz	144	95	125	158	111	97	1009	19058	4414	1143452	123591
wołomiński	Wołomin	34	29	46	35	15	12	240	4520	1047	271217	29315
wołomiński	Zielonka	0	0	0	0	0	0	2	26	6	1534	166
wyszkowski	Brańszczyk	241	123	196	74	186	117	1639	27117	6281	1627035	175859
wyszkowski	Długosiodło	364	189	298	118	255	174	2171	37082	8589	2224947	240485
wyszkowski	Rząśnik	647	305	373	130	483	381	3566	59870	13867	3592190	388264
wyszkowski	Somianka	709	555	980	255	392	301	2370	52938	12261	3176277	343310
wyszkowski	Wyszków	263	228	421	147	234	156	1255	26844	6217	1610625	174085
wyszkowski	Zabrodzie	295	165	250	129	200	189	1831	32162	7449	1929691	208572
zwoleński	Kazanów	473	216	429	100	265	255	2112	38525	8923	2311480	249838
zwoleński	Policzna	463	316	519	96	263	207	1611	32836	7605	1970178	212948
zwoleński	Przyłęk	214	147	183	58	134	100	975	17879	4141	1072753	115949
zwoleński	Tczów	427	203	379	73	201	230	1463	28597	6623	1715830	185456
zwoleński	Zwoleń	356	158	339	116	218	206	1460	28430	6585	1705823	184375
żuromiński	Bieżuń	1262	467	718	479	993	730	7461	126743	29355	7604586	821946
żuromiński	Kuczbork-Osada	622	369	564	272	525	358	3403	62403	14453	3744208	404695
żuromiński	Lubowidz	802	619	775	444	654	460	3735	74722	17307	4483326	484583
żuromiński	Lutocin	1362	481	720	477	978	866	7688	131207	30389	7872429	850896
żuromiński	Siemiątkowo	358	184	285	69	312	280	1971	34788	8057	2087306	225608
żuromiński	Siemiątkowo Koziebrodzkie	564	193	307	264	377	344	3418	58328	13509	3499675	378264
żuromiński	Żuromin	855	466	664	463	653	472	4491	83223	19275	4993363	539710
żyrardowski	Mszczonów	62	74	103	144	42	30	489	10749	2490	644955	69710
żyrardowski	Puszcza Mariańska	73	49	64	73	61	36	443	8552	1981	513104	55459
żyrardowski	Radziejowice	31	19	22	27	30	15	169	3268	757	196109	21197

żyrardowski	Wiskitki	185	96	163	61	113	118	765	14897	3450	893820	96609
żyrardowski	Żyrardów	0	0	0	2	0	0	3	74	17	4447	481

PODLASKIE Voivodeship												
**Province**	**Community**	**Calves**	**Bulls 6-12 months**	**Bulls 12-24 months**	**Bulls >24 months**	**Heifers 6-12 months**	**Heifers 12-18 months**	**Dairy cows**	**Manure mass (tons)**	**Slurry volume (m^3)**	**Amount of biogas from manure (m^3)**	**Amount of biogas from slurry (m^3)**
augustowski	Augustów	2736	963	1317	166	1981	1775	13553	210894	69859	12653669	1956062
augustowski	Bargłów Kościelny	2512	710	1006	150	1823	1719	13766	207772	68825	12466307	1927098
augustowski	Lipsk	1123	564	891	106	743	579	4553	77352	25623	4641118	717445
augustowski	Nowinka	473	273	363	44	318	230	1788	30906	10238	1854335	286652
augustowski	Płaska	53	60	64	11	68	45	373	6271	2077	376274	58166
augustowski	Sztabin	1749	764	1062	143	1209	981	8180	130589	43258	7835354	1211225
białostocki	Choroszcz	521	332	527	76	381	255	2218	38953	12903	2337194	361294
białostocki	Czarna Białostocka	217	144	343	35	87	91	542	12496	4139	749772	115903
białostocki	Dobrzyniewo Duże	408	157	283	52	346	305	2566	39905	13219	2394325	370126
białostocki	Gródek	398	186	221	78	335	276	2343	36836	12202	2210135	341653
białostocki	Juchnowiec Kościelny	605	394	605	83	358	245	2180	39560	13104	2373599	366922
białostocki	Łapy	1417	852	1196	149	583	710	4127	78139	25884	4688337	724744
białostocki	Michałowo	519	201	313	107	382	340	3002	47182	15629	2830938	437619
białostocki	Poświętne	2651	1042	1659	340	1493	1471	11457	187376	62069	11242544	1737924
białostocki	Supraśl	27	8	15	3	23	7	145	2165	717	129870	20076
białostocki	Suraż	323	247	273	25	213	147	1221	21368	7078	1282085	198190
białostocki	Turośń Kościelna	311	272	441	103	162	85	743	17840	5910	1070428	165472
białostocki	Tykocin	1875	865	1467	185	1199	1090	8765	143820	47641	8629209	1333942
białostocki	Wasilków	33	29	36	7	21	8	155	2643	875	158562	24511
białostocki	Zabłudów	641	368	623	111	397	378	2942	50750	16811	3044973	470706
białostocki	Zawady	1032	451	650	89	807	737	6321	97170	32188	5830207	901260
Białystok	Białystok	19	14	14	4	14	13	87	1507	499	90437	13980
bielski	Bielsk Podlaski	1081	595	987	193	674	538	5293	88022	29158	5281349	816415

bielski	Boćki	1400	732	1016	171	958	705	7482	117930	39064	7075778	1093806
bielski	Brańsk	2327	1018	1530	238	1652	1528	11893	190109	62974	11406548	1763276
bielski	Orla	130	114	128	31	128	54	861	13722	4546	823340	127276
bielski	Rudka	285	106	149	24	277	211	1676	25895	8578	1553720	240181
bielski	Wyszki	1503	739	1143	161	818	776	6412	106515	35283	6390887	987932
grajewski	Grajewo	2377	886	1317	196	1618	1458	11620	182894	60584	10973648	1696356
grajewski	Radziłów	6746	2348	3390	495	4680	4183	33854	525364	174028	31521857	4872792
grajewski	Rajgród	1486	326	514	72	1156	932	8429	124405	41209	7464271	1153861
grajewski	Szczuczyn	1482	644	941	145	1111	932	7974	125247	41488	7514806	1161673
grajewski	Wąsosz	1005	481	733	123	832	698	5447	87813	29088	5268806	814476
hajnowski	Czeremcha	38	17	18	7	23	16	124	2147	711	128825	19914
hajnowski	Czyże	232	131	166	30	161	80	991	16247	5382	974801	150689
hajnowski	Dubicze Cerkiewne	79	86	134	12	76	66	485	8678	2875	520670	80488
hajnowski	Hajnówka	441	268	434	54	306	264	2141	36205	11993	2172324	335808
hajnowski	Kleszczele	167	362	162	34	174	68	623	12501	4141	750060	115948
hajnowski	Narew	288	188	228	63	222	145	1789	28137	9320	1688204	260970
hajnowski	Narewka	109	67	62	6	73	51	483	7691	2548	461484	71338
kolneński	Grabowo	2013	1125	1686	319	1242	1078	8426	145332	48141	8719898	1347962
kolneński	Kolno	3963	1899	2485	452	2705	2385	19248	309416	102495	18564941	2869853
kolneński	Mały Płock	1613	865	1194	162	932	748	6216	105461	34934	6327664	978159
kolneński	Stawiski	2415	1160	1679	177	1307	1254	9072	153414	50819	9204848	1422927
kolneński	Turośl	1482	381	434	116	1698	937	12770	178726	59203	10723532	1657692
Łomża	Łomża	25	46	43	11	27	20	155	2961	981	177666	27464
łomżyński	Jedwabne	1912	1095	1430	161	1195	1217	8258	139071	46068	8344273	1289896
łomżyński	Łomża	2079	730	1018	151	1194	1206	8916	141725	46947	8503484	1314507
łomżyński	Miastkowo	914	481	688	78	490	492	3667	61860	20491	3711626	573760
łomżyński	Nowogród	845	428	536	66	454	405	3392	55612	18422	3336722	515806
łomżyński	Piątnica	1802	1120	1534	318	852	931	5752	108509	35944	6510520	1006426
łomżyński	Przytuły	951	434	727	179	566	531	4520	74729	24754	4483746	693118

łomżyński	Śniadowo	3244	577	877	224	2110	2402	17782	264468	87606	15868090	2452962
łomżyński	Wizna	2033	785	1061	156	1138	1128	8041	130873	43352	7852367	1213855
łomżyński	Zbójna	815	262	453	90	875	412	5909	86392	28618	5183523	801293
moniecki	Goniądz	1440	526	838	92	894	784	6602	104175	34508	6250491	966229
moniecki	Jasionówka	936	637	892	102	567	445	3329	60592	20071	3635499	561992
moniecki	Jaświły	2192	1114	1668	202	1240	1160	7769	136361	45170	8181638	1264755
moniecki	Knyszyn	796	464	607	75	476	378	2697	47957	15886	2877400	444802
moniecki	Krypno	1156	434	586	81	786	750	6053	93412	30943	5604709	866401
moniecki	Mońki	1695	844	1373	142	908	856	6611	112615	37304	6756888	1044510
moniecki	Trzcianne	1391	759	1070	130	780	737	5630	95254	31553	5715259	883491
sejneński	Giby	378	291	387	40	280	182	1161	22831	7563	1369854	211758
sejneński	Krasnopol	994	609	815	98	688	466	3988	68344	22639	4100623	633893
sejneński	Puńsk	1963	1012	1281	209	1320	1173	8794	144707	47935	8682411	1342167
sejneński	Sejny	1597	1128	1596	175	1063	701	5748	104860	34735	6291619	972587
siemiatycki	Drohiczyn	1739	700	1114	146	971	928	6772	112606	37301	6756383	1044432
siemiatycki	Dziadkowice	686	302	414	62	441	416	3139	50650	16778	3039026	469786
siemiatycki	Grodzisk	1509	526	713	131	1096	963	7890	121678	40306	7300662	1128570
siemiatycki	Mielnik	81	49	64	7	49	45	419	6699	2219	401938	62133
siemiatycki	Milejczyce	374	141	191	42	248	210	1750	27663	9164	1659800	256579
siemiatycki	Nurzec-Stacja	147	87	131	40	80	62	590	10484	3473	629021	97237
siemiatycki	Perlejewo	1345	424	688	130	796	879	6328	100101	33159	6006070	928446
siemiatycki	Siemiatycze	1038	491	644	126	615	562	4289	71101	23552	4266070	659468
sokólski	Dąbrowa Białostocka	1981	1338	1826	286	1210	868	6951	126976	42061	7618579	1177715
sokólski	Janów	1709	976	1585	240	868	830	5526	103372	34242	6202299	958780
sokólski	Korycin	1789	950	1575	191	924	797	5916	107072	35468	6424336	993103
sokólski	Krynki	54	19	24	25	54	31	292	4818	1596	289081	44687
sokólski	Kuźnica	372	244	403	75	214	189	1445	26635	8823	1598091	247040
sokólski	Nowy Dwór	705	493	806	145	455	318	2211	44496	14739	2669745	412701

sokólski	Sidra	684	552	739	128	489	330	2623	48929	16208	2935721	453817
sokólski	Sokółka	1006	788	934	230	696	481	4256	75768	25098	4546067	702752
sokólski	Suchowola	2636	1302	2051	332	1363	1445	8966	161271	53422	9676280	1495803
sokólski	Szudziałowo	319	213	277	56	228	166	1550	25988	8609	1559271	241039
suwalski	Bakałarzewo	1075	633	795	78	774	554	4405	74214	24583	4452819	688337
suwalski	Filipów	1020	859	764	106	946	454	4919	80937	26810	4856199	750693
suwalski	Jeleniewo	1008	772	998	121	739	407	3822	68725	22765	4123476	637426
suwalski	Przerośl	836	646	618	122	828	389	4377	71141	23566	4268449	659836
suwalski	Raczki	1176	735	741	85	861	659	5073	83711	27729	5022654	776425
suwalski	Rutka-Tartak	816	536	551	142	765	493	4633	74249	24595	4454946	688666
suwalski	Suwałki	753	681	676	111	610	447	3476	60436	20020	3626163	560549
suwalski	Szypliszki	1464	899	1182	167	1036	669	5869	100730	33367	6043809	934280
suwalski	Wiżajny	934	483	437	40	826	437	5177	77677	25731	4660619	720460
Suwałki	Suwałki	26	50	42	25	37	18	140	3040	1007	182388	28194
wysokomazowiecki	Ciechanowiec	1553	466	740	105	1067	1027	7746	120143	39798	7208604	1114339
wysokomazowiecki	Czyżew	2233	403	835	176	1416	1563	11059	169465	56136	10167898	1571800
wysokomazowiecki	Czyżew-Osada	38	8	15	4	39	33	366	5164	1711	309843	47897
wysokomazowiecki	Klukowo	2079	436	703	140	1376	1481	10676	161898	53629	9713875	1501615
wysokomazowiecki	Kobylin-Borzymy	1922	786	1101	160	1306	1342	9731	154096	51045	9245759	1429251
wysokomazowiecki	Kulesze Kościelne	1781	325	595	120	1319	1344	10313	153702	50914	9222149	1425602
wysokomazowiecki	Nowe Piekuty	1721	320	467	123	1278	1286	10197	150297	49786	9017846	1394020
wysokomazowiecki	Sokoły	2016	518	615	95	1387	1434	9696	148290	49122	8897420	1375404
wysokomazowiecki	Szepietowo	2233	445	593	99	1597	1776	11681	176168	58356	10570088	1633972
wysokomazowiecki	Wysokie Mazowieckie	2487	549	806	132	1597	1703	12078	183672	60842	11020290	1703567
zambrowski	Kołaki Kościelne	1344	230	445	39	845	948	7032	104832	34726	6289925	972325
zambrowski	Rutki	2158	814	1189	179	1380	1313	10029	159356	52787	9561369	1478040
zambrowski	Szumowo	1639	742	1058	128	1068	1014	7616	123053	40762	7383205	1141329
zambrowski	Zambrów	3053	951	1392	165	1929	2076	13926	219314	72648	13158840	2034153

Wielkopolskie Voivodeship												
**Province**	**Community**	**Calves**	**Bulls 6-12 months**	**Bulls 12-24 months**	**Bulls >24 months**	**Heifers 6-12 months**	**Heifers 12-18 months**	**Dairy Cows**	**Manure mass (tones)**	**Slurry volume (m^3)**	**Amount of biogas from manure (m^3)**	**Amount of biogas from slurry (m^3)**
chodzieski	Budzyń	572	659	1097	181	281	184	1070	38662	2770	2319736	77569
chodzieski	Chodzież	417	378	609	121	231	173	1083	30224	2166	1813468	60640
chodzieski	Margonin	701	578	916	125	214	144	1115	35313	2530	2118809	70850
chodzieski	Szamocin	325	280	396	106	143	157	687	20690	1483	1241392	41511
czarnkowsko-trzcianecki	Czarnków	1121	552	1034	122	525	536	3447	76371	5472	4582289	153226
czarnkowsko-trzcianecki	Drawsko	322	137	258	53	222	162	1615	31049	2225	1862963	62295
czarnkowsko-trzcianecki	Krzyż Wielkopolski	176	169	157	28	155	91	1101	20814	1491	1248865	41760
czarnkowsko-trzcianecki	Lubasz	519	207	432	78	285	239	1766	37544	2690	2252646	75326
czarnkowsko-trzcianecki	Połajewo	1479	977	1575	149	718	770	4484	103901	7445	6234033	208458
czarnkowsko-trzcianecki	Trzcianka	307	159	270	47	121	90	849	18973	1360	1138387	38066
czarnkowsko-trzcianecki	Wieleń	582	281	396	93	415	361	2889	55814	3999	3348841	111981
gnieźnieński	Czerniejewo	542	189	343	64	140	126	1320	27969	2004	1678136	56115
gnieźnieński	Gniezno	1222	1060	1532	296	443	402	2196	68211	4888	4092676	136854
gnieźnieński	Kiszkowo	150	212	377	92	55	27	289	12009	860	720519	24093
gnieźnieński	Kłecko	669	487	775	101	168	202	1152	33763	2419	2025797	67740
gnieźnieński	Łubowo	576	657	973	218	171	117	759	32328	2316	1939661	64860

gnieźnieński	Mieleszyn	388	559	826	115	120	63	304	20471	1467	1228281	41072
gnieźnieński	Niechanowo	1099	841	1354	161	534	527	2684	71055	5091	4263283	142559
gnieźnieński	Trzemeszno	1362	656	1159	106	712	669	3402	80107	5740	4806448	160721
gnieźnieński	Witkowo	1422	895	1561	189	521	488	2819	76312	5468	4578745	153107
gostyński	Borek Wielkopolski	2777	2942	4254	609	1068	944	5919	178643	12801	10718571	358415
gostyński	Gostyń	2017	1078	1500	192	892	907	5098	116527	8350	6991620	233791
gostyński	Krobia	2851	1712	2378	518	1424	1377	6275	161890	11600	9713380	324803
gostyński	Pępowo	1912	1173	1791	308	726	813	5109	120711	8649	7242683	242186
gostyński	Piaski	1697	782	1392	219	904	946	4588	107123	7676	6427401	214924
gostyński	Pogorzela	2259	889	1167	255	904	1069	6012	128607	9215	7716409	258027
gostyński	Poniec	1541	920	1552	182	646	616	3305	85341	6115	5120458	171221
grodziski	Granowo	661	569	1010	135	270	203	1108	37271	2671	2236247	74777
grodziski	Grodzisk Wielkopolski	948	681	1127	168	345	381	2101	56835	4072	3410080	114029
grodziski	Kamieniec	1425	1127	1924	261	567	571	2977	86269	6182	5176145	173084
grodziski	Rakoniewice	1151	1009	1561	295	263	313	1546	57117	4093	3427034	114596
grodziski	Wielichowo	1660	1791	2971	527	394	328	1794	86485	6197	5189085	173516
jarociński	Jaraczewo	1511	855	1472	237	623	676	3812	92987	6663	5579195	186561
jarociński	Jarocin	964	766	1193	151	509	519	1996	58422	4186	3505312	117213
jarociński	Kotlin	481	528	676	146	123	123	587	24172	1732	1450305	48496
jarociński	Żerków	1655	1449	2086	289	689	682	3250	96209	6894	5772563	193027
kaliski	Blizanów	835	565	867	148	512	421	2289	56583	4054	3395005	113525
kaliski	Brzeziny	421	330	569	122	238	167	706	24187	1733	1451244	48528
kaliski	Ceków-Kolonia	556	457	783	117	168	212	961	31199	2236	1871931	62595
kaliski	Godziesze Wielkie	727	709	1133	301	257	206	921	40035	2869	2402114	80324
kaliski	Kalisz	0	0	0	0	0	0	0	0	0	0	0
kaliski	Koźminek	414	409	675	100	209	169	839	27071	1940	1624242	54313
kaliski	Lisków	988	774	1171	197	457	396	2246	61109	4379	3666566	122605

kaliski	Mycielin	636	421	699	147	298	296	1629	41878	3001	2512677	84021
kaliski	Opatówek	396	367	552	124	155	122	738	23727	1700	1423614	47604
kaliski	Stawiszyn	315	215	365	52	130	135	636	17930	1285	1075782	35973
kaliski	Szczytniki	512	728	1098	141	324	220	952	36957	2648	2217414	74147
kaliski	Żelazków	785	596	886	142	339	294	1708	46250	3314	2775014	92793
Kalisz	Kalisz	64	55	96	20	21	25	137	4188	300	251276	8402
kępiński	Baranów	569	608	1003	206	103	102	470	27631	1980	1657851	55436
kępiński	Bralin	178	107	214	43	110	78	637	14557	1043	873403	29205
kępiński	Kępno	597	395	865	165	194	197	1057	34219	2452	2053128	68654
kępiński	Łęka Opatowska	466	406	722	145	111	106	579	23764	1703	1425835	47678
kępiński	Perzów	298	292	404	46	147	124	877	21925	1571	1315485	43988
kępiński	Rychtal	77	50	161	17	24	36	190	5767	413	346039	11571
kępiński	Trzcinica	291	176	284	66	75	99	525	14844	1064	890613	29781
kolski	Babiak	1076	517	777	131	555	575	3533	75175	5387	4510502	150825
kolski	Chodów	818	258	415	135	448	376	3727	69514	4981	4170827	139467
kolski	Dąbie	829	538	796	118	546	405	2949	64407	4615	3864418	129221
kolski	Grzegorzew	637	386	437	71	327	284	1902	41106	2945	2466342	82471
kolski	Kłodawa	1166	689	989	151	583	535	4109	86778	6218	5206679	174105
kolski	Koło	924	565	832	112	399	409	2722	61202	4385	3672118	122791
kolski	Kościelec	556	402	553	106	344	296	2129	46460	3329	2787612	93214
kolski	Olszówka	479	226	327	63	225	214	1559	32558	2333	1953455	65321
kolski	Osiek Mały	311	287	404	84	102	70	492	16401	1175	984051	32905
kolski	Przedecz	867	496	793	156	426	423	2484	58105	4163	3486308	116578
Konin	Konin	91	29	63	13	62	44	248	5591	401	335448	11217
koniński	Golina	472	256	489	70	346	262	1652	37042	2654	2222539	74319
koniński	Grodziec	662	591	1093	265	247	282	1420	46146	3307	2768736	92583
koniński	Kazimierz Biskupi	118	164	198	19	52	38	236	7600	545	456004	15248
koniński	Kleczew	367	343	514	69	104	111	663	20623	1478	1237377	41376
koniński	Konin	0	0	0	0	0	0	0	0	0	0	0

koniński	Kramsk	1165	551	785	110	790	682	4704	94074	6741	5644448	188743
koniński	Krzymów	432	294	514	96	225	198	1106	28904	2071	1734250	57991
koniński	Rychwał	886	882	1432	239	451	379	1913	60203	4314	3612160	120786
koniński	Rzgów	667	506	812	105	270	240	1163	35366	2534	2121969	70956
koniński	Skulsk	353	209	338	48	127	108	736	18701	1340	1122074	37521
koniński	Sompolno	701	394	750	109	280	300	1733	43174	3094	2590443	86621
koniński	Stare Miasto	694	783	1036	231	319	200	1328	43771	3136	2626272	87819
koniński	Ślesin	684	263	479	78	340	335	2443	49636	3557	2978161	99586
koniński	Wierzbinek	846	564	885	204	422	325	2476	59108	4235	3546479	118590
koniński	Wilczyn	476	374	734	96	212	184	1119	31815	2280	1908914	63832
kościański	Czempiń	1422	827	1476	277	663	547	3300	84933	6086	5095988	170403
kościański	Kościan	2283	1461	2201	357	743	803	5382	132224	9474	7933436	265284
kościański	Krzywiń	2813	1856	3466	499	993	950	4602	143917	10312	8634994	288743
kościański	Śmigiel	3411	2038	3360	574	1708	1714	7533	199735	14312	11984091	400733
krotoszyński	Kobylin	1982	1356	1997	211	1158	987	5009	124458	8918	7467482	249703
krotoszyński	Koźmin Wielkopolski	2803	1753	2822	307	931	1206	5213	143643	10293	8618595	288195
krotoszyński	Krotoszyn	4554	2200	3945	578	2025	1841	10297	251755	18039	15105271	505101
krotoszyński	Rozdrażew	2508	1520	2597	395	940	966	5106	136992	9816	8219517	274850
krotoszyński	Sulmierzyce	204	169	242	27	98	104	528	13570	972	814214	27226
krotoszyński	Zduny	1269	428	667	114	599	636	3401	72619	5203	4357148	145697
leszczyński	Krzemieniewo	1588	886	1579	190	609	543	2873	78624	5634	4717451	157745
leszczyński	Lipno	566	452	806	59	173	135	973	29620	2122	1777211	59428
leszczyński	Osieczna	1547	897	1635	243	321	380	1897	63298	4536	3797884	126996
leszczyński	Rydzyna	1115	769	1394	233	405	441	2380	66814	4788	4008846	134051
leszczyński	Święciechowa	985	542	781	125	530	493	2058	52976	3796	3178576	106287
leszczyński	Wijewo	716	633	757	119	434	293	1593	43091	3088	2585446	86454
leszczyński	Włoszakowice	737	729	1000	160	174	215	764	33331	2388	1999832	66872
Leszno	Leszno	26	26	13	14	4	0	253	4212	302	252728	8451

międzychodzki	Chrzypsko Wielkie	422	478	833	102	231	151	728	27530	1973	1651829	55235
międzychodzki	Kwilcz	202	308	420	59	92	44	381	14023	1005	841374	28134
międzychodzki	Międzychód	492	543	873	346	234	182	1002	37542	2690	2252518	75321
międzychodzki	Sieraków	359	387	500	64	194	161	694	21930	1571	1315784	43998
nowotomyski	Kuślin	399	248	537	61	120	111	745	21624	1549	1297421	43384
nowotomyski	Lwówek	778	531	1039	98	319	248	1362	41289	2959	2477317	82838
nowotomyski	Miedzichowo	173	124	212	19	51	30	232	7554	541	453228	15155
nowotomyski	Nowy Tomyśl	481	295	627	91	206	200	1052	29373	2105	1762393	58932
nowotomyski	Opalenica	849	714	1041	108	329	268	1759	48366	3466	2901933	97037
nowotomyski	Zbąszyń	432	308	543	90	166	148	656	21948	1573	1316851	44034
obornicki	Oborniki	1174	895	1437	124	500	445	1967	60368	4326	3622067	121117
obornicki	Rogoźno	526	397	764	158	197	163	761	28198	2021	1691887	56575
obornicki	Ryczywół	1054	1153	2137	338	456	378	1847	70791	5072	4247439	142029
ostrowski	Nowe Skalmierzyce	1091	648	1246	218	358	381	2067	58922	4222	3535333	118217
ostrowski	Odolanów	871	530	992	231	372	345	2091	55307	3963	3318392	110963
ostrowski	Ostrów Wielkopolski	1385	1132	1738	286	445	371	2575	76064	5450	4563841	152609
ostrowski	Przygodzice	999	558	933	233	443	428	2487	61953	4439	3717171	124297
ostrowski	Raszków	1813	1353	2446	455	574	628	3003	98923	7088	5935354	198471
ostrowski	Sieroszewice	1434	1190	1935	480	454	413	2379	80253	5750	4815160	161013
ostrowski	Sośnie	369	260	351	86	185	170	981	24300	1741	1457991	48753
ostrzeszowski	Czajków	303	221	351	96	107	101	600	17649	1265	1058956	35410
ostrzeszowski	Doruchów	666	677	1400	215	237	264	1192	45528	3262	2731668	91343
ostrzeszowski	Grabów nad Prosną	1396	1003	1648	285	575	596	3070	84656	6066	5079333	169846
ostrzeszowski	Kobyla Góra	492	356	603	88	284	250	1540	37173	2664	2230397	74582
ostrzeszowski	Kraszewice	406	357	385	69	146	187	933	24185	1733	1451116	48523
ostrzeszowski	Mikstat	759	526	1024	176	251	245	1352	42143	3020	2528606	84553

ostrzeszowski	Ostrzeszów	504	552	876	141	323	223	1728	44736	3206	2684180	89756
pilski	Białośliwie	604	288	530	230	257	324	1678	41812	2996	2508748	83889
pilski	Kaczory	602	353	684	95	265	292	1627	40093	2873	2405573	80439
pilski	Łobżenica	1621	1199	1776	472	887	742	4687	117170	8396	7030225	235082
pilski	Miasteczko Krajeńskie	558	192	467	117	320	303	1855	40884	2930	2453061	82027
pilski	Piła	23	3	8	0	10	12	78	1443	103	86563	2895
pilski	Szydłowo	23	21	23	18	22	20	157	3316	238	198963	6653
pilski	Ujście	299	173	293	63	143	162	1039	23215	1663	1392909	46577
pilski	Wyrzysk	1864	1303	2153	387	932	850	4846	124418	8915	7465090	249623
pilski	Wysoka	1090	495	855	258	629	600	3387	77053	5521	4623158	154592
pleszewski	Chocz	205	138	209	75	76	93	330	10983	787	658981	22035
pleszewski	Czermin	482	510	650	154	208	181	822	28329	2030	1699744	56837
pleszewski	Dobrzyca	1169	795	1292	137	388	402	1867	56146	4023	3368784	112648
pleszewski	Gizałki	504	325	578	123	211	150	875	26563	1903	1593751	53293
pleszewski	Gołuchów	759	613	1032	382	284	308	1661	51968	3724	3118106	104265
pleszewski	Pleszew	704	546	919	229	288	237	1106	38537	2761	2312220	77318
Poznań	Poznań	105	43	96	530	54	53	342	17766	1273	1065960	35644
poznański	Buk	459	640	1076	155	202	137	1079	36838	2640	2210283	73909
poznański	Czerwonak	35	21	20	2	13	10	51	1327	95	79645	2663
poznański	Dopiewo	76	65	133	34	27	17	162	5247	376	314821	10527
poznański	Kleszczewo	424	467	904	177	120	139	772	29807	2136	1788400	59802
poznański	Komorniki	77	118	120	19	8	2	28	2869	206	172144	5756
poznański	Kostrzyn	899	548	1015	130	378	392	2424	58993	4227	3539604	118360
poznański	Kórnik	818	531	982	129	233	286	1779	47344	3392	2840651	94988
poznański	Luboń	0	0	0	0	0	0	9	128	9	7687	257
poznański	Mosina	507	591	1004	192	90	84	607	28848	2067	1730876	57878
poznański	Murowana Goślina	90	76	86	23	20	31	172	4841	347	290479	9713

poznański	Pobiedziska	382	244	455	100	107	173	870	23844	1709	1430618	47838
poznański	Puszczykowo	0	2	2	0	0	0	3	74	5	4441	149
poznański	Rokietnica	208	86	113	24	136	137	585	13045	935	782697	26172
poznański	Stęszew	848	727	1175	151	401	397	1794	53077	3803	3184597	106489
poznański	Suchy Las	56	62	79	11	50	32	124	3846	276	230778	7717
poznański	Swarzędz	155	83	124	17	60	61	366	8597	616	515834	17249
poznański	Tarnowo Podgórne	135	145	354	52	7	3	42	6637	476	398224	13316
rawicki	Bojanowo	1670	751	1290	158	866	873	3927	94153	6746	5649188	188902
rawicki	Jutrosin	2320	2109	3835	601	600	562	3070	122274	8761	7336463	245322
rawicki	Miejska Górka	2242	2187	3322	548	438	609	3089	115657	8287	6939392	232044
rawicki	Pakosław	1920	1394	2248	455	576	624	3066	97931	7017	5875866	196481
rawicki	Rawicz	1647	1238	2209	293	559	466	2396	81600	5847	4896000	163716
słupecki	Lądek	715	752	1446	180	264	198	808	39677	2843	2380590	79604
słupecki	Orchowo	306	202	460	97	86	66	434	15984	1145	959069	32070
słupecki	Ostrowite	383	330	435	67	150	89	605	18782	1346	1126942	37683
słupecki	Powidz	45	43	94	6	22	13	72	2737	196	164201	5491
słupecki	Słupca	906	712	1193	164	305	306	1761	51684	3703	3101066	103696
słupecki	Strzałkowo	482	418	569	65	182	207	935	27034	1937	1622021	54238
słupecki	Zagórów	1176	870	1475	197	591	507	2459	70251	5034	4215069	140946
szamotulski	Duszniki	673	888	1216	155	334	204	1208	43111	3089	2586685	86495
szamotulski	Kaźmierz	237	231	390	16	125	116	671	17659	1265	1059554	35430
szamotulski	Obrzycko	231	385	508	52	140	146	528	18735	1342	1124124	37589
szamotulski	Ostroróg	296	301	407	49	126	109	640	18419	1320	1105120	36954
szamotulski	Pniewy	639	627	619	99	153	154	829	27272	1954	1636327	54717
szamotulski	Szamotuły	1059	736	1160	174	446	398	2134	58897	4220	3533796	118166
szamotulski	Wronki	600	418	613	59	239	189	1228	31876	2284	1912586	63954
średzki	Dominowo	428	177	430	88	190	194	1171	28010	2007	1680613	56198
średzki	Krzykosy	496	693	816	183	116	119	719	29044	2081	1742620	58271

średzki	Nowe Miasto nad Wartą	745	405	669	134	334	320	1997	47107	3375	2826430	94512
średzki	Środa Wielkopolska	986	678	1001	194	435	526	2175	59075	4233	3544515	118524
średzki	Zaniemyśl	524	471	596	67	194	151	1501	35168	2520	2110054	70557
śremski	Brodnica	1265	531	853	99	488	603	2954	67657	4848	4059409	135741
śremski	Dolsk	2438	755	1087	136	301	281	1546	50012	3584	3000710	100340
śremski	Książ Wielkopolski	1166	887	1239	186	319	229	1650	51552	3694	3093123	103430
śremski	Śrem	1362	1054	1787	262	422	466	2268	72375	5186	4342501	145208
turecki	Brudzew	433	292	425	69	250	217	1558	34104	2444	2046210	68423
turecki	Dobra	751	471	631	56	346	333	2113	47265	3387	2835911	94829
turecki	Kawęczyn	1218	609	1071	194	672	623	4000	88169	6318	5290125	176895
turecki	Malanów	799	558	661	53	463	332	2385	52381	3753	3142874	105094
turecki	Przykona	474	335	489	36	249	185	1336	30917	2215	1855020	62029
turecki	Tuliszków	677	506	759	86	398	301	1680	43006	3082	2580364	86284
turecki	Turek	641	381	851	137	363	309	1835	46608	3340	2796452	93510
turecki	Władysławów	358	285	455	46	208	154	790	21967	1574	1318047	44074
wągrowiecki	Damasławek	953	835	1287	389	319	271	1393	52287	3747	3137237	104905
wągrowiecki	Gołańcz	1020	1084	1761	284	427	205	1220	54046	3873	3242761	108434
wągrowiecki	Mieścisko	619	404	954	124	212	182	1146	35674	2556	2140417	71573
wągrowiecki	Skoki	353	282	486	47	84	124	476	16976	1216	1018557	34059
wągrowiecki	Wapno	346	171	314	33	114	151	758	18699	1340	1121946	37516
wągrowiecki	Wągrowiec	1884	1324	2361	401	690	794	3859	111477	7988	6688628	223659
wolsztyński	Przemęt	2160	1778	3108	629	744	667	3874	126054	9032	7563226	252905
wolsztyński	Siedlec	1360	858	1745	403	437	429	2174	72170	5171	4330202	144796
wolsztyński	Wolsztyn	1257	1022	1895	238	453	333	1901	66159	4741	3969558	132737
wrzesiński	Kołaczkowo	867	714	1084	214	413	387	2170	58571	4197	3514237	117512
wrzesiński	Miłosław	724	322	452	71	216	247	1512	34742	2489	2084516	69704
wrzesiński	Nekla	310	272	318	57	163	130	922	21882	1568	1312923	43902

wrzesiński	Pyzdry	781	606	958	232	325	274	1530	46077	3302	2764594	92444
wrzesiński	Września	1383	580	1030	173	738	625	3641	82706	5926	4962364	165935
złotowski	Jastrowie	106	58	54	17	65	50	427	8353	599	501186	16759
złotowski	Krajenka	457	473	675	221	154	170	920	30777	2205	1846607	61748
złotowski	Lipka	457	303	452	165	255	182	1544	35846	2569	2150752	71918
złotowski	Okonek	74	64	40	126	34	23	238	7193	515	431577	14431
złotowski	Tarnówka	38	34	37	18	27	13	240	4686	336	281170	9402
złotowski	Zakrzewo	763	445	791	273	314	319	2290	55550	3980	3332997	111451
złotowski	Złotów	914	547	894	216	483	434	3151	70633	5061	4237959	141712
